# Robotic Surgery in the Management of Renal Tumors During Pregnancy: A Narrative Review

**DOI:** 10.3390/cancers17040574

**Published:** 2025-02-08

**Authors:** Lucio Dell’Atti, Viktoria Slyusar

**Affiliations:** 1 Department of Urology, University-Hospital of Marche, 60126 Ancona, Italy; 2 Pain Therapy Center, Division of Anesthesia and Intensive Care, University-Hospital of Marche, 60126 Ancona, Italy; viktoria.slyusar@ospedaliriuniti.marche.it

**Keywords:** pregnancy, renal tumor, robotic surgery, management, renal cell carcinoma, laparoscopic surgery

## Abstract

Renal masses are uncommon during pregnancy; they represent the most frequently encountered urological cancer in pregnant patients and require careful surgical planning. The introduction of robotic surgical systems aims to address these challenges by simplifying intra-corporeal suturing and reducing technical complexity. Robot-assisted laparoscopic renal surgery offers potential benefits over both open surgery and conventional laparoscopy, providing greater precision and reduced invasiveness, particularly in tumor excision and suturing. Although urological tumors during pregnancy are rare, early detection significantly improves outcomes by enabling intervention before the tumor advances and while the uterus remains relatively small. The decision regarding the timing and necessity of surgery in pregnant patients requires a careful assessment of maternal health, fetal development, and the progression of the disease. Risks for adverse pregnancy outcomes should be explained, and the patient’s decision about pregnancy termination should be considered. Radical nephrectomy or nephron-sparing surgery are essential treatments for the management of renal tumors. Effective management demands close collaboration between a multidisciplinary team and the patient to ensure individualized care. The aim of this review was to evaluate the renal tumors during pregnancy in terms of epidemiology, risk factors, diagnosis and the safety of a robot-assisted laparoscopic approach in the management of these tumors.

## Simple Summary

Managing renal masses during pregnancy is a complex and challenging topic. Any solid renal tumor identified during pregnancy should be presumed to be renal cell carcinoma until proven otherwise. There is no universal agreement on the optimal timing for treatments. However, the decision regarding the timing and necessity of surgery in pregnant patients requires a careful assessment of maternal health, fetal development, and the progression of the disease. Laparoscopic techniques have transformed minimally invasive surgery. Today, robotic surgery, suitable even during pregnancy, offers advantages like enhanced visualization and precision, particularly when operating alongside a gravid uterus.

## 1. Introduction

Robotic surgery during pregnancy is becoming increasingly prevalent due to the advantages of minimally invasive procedures [1]. Although renal cell tumors are uncommon during pregnancy, they represent the most frequently encountered urological cancer in pregnant patients and require careful surgical planning [1,2]. For localized renal tumors, partial nephrectomy (PN) is often recommended to preserve renal function. This approach is particularly suitable for patients with exophytic renal masses measuring less than 4 cm [3]. However, current guidelines from both the American Urological Association and the European Association of Urology recommend PN for all cT1a–b renal tumors. This change in guideline recommendations has come following the publication of multiple retrospective studies demonstrating that PN is not inferior to radical nephrectomy (RN) for clinically T1a–b renal tumors. PN can additionally be used in the management of benign renal pathologies like angiomyolipoma and oncocytoma [3,4].

Laparoscopic partial nephrectomy (LPN) is gaining recognition as a minimally invasive and effective treatment [4]. However, the procedure presents challenges, such as the prolonged time required for tumor excision, hemostasis, and suturing, even for skilled surgeons [5]. The introduction of robotic surgical systems, such as the da Vinci platform, aims to address these challenges by simplifying intracorporeal suturing and reducing technical complexity [6]. Robot-assisted laparoscopic partial nephrectomy (RLPN) offers potential benefits over both open surgery and conventional laparoscopy, providing greater precision and reduced invasiveness, particularly in tumor excision and suturing [7]. Although urological tumors during pregnancy are rare, early detection significantly improves outcomes by enabling intervention before the tumor advances and while the uterus remains relatively small [8]. The aim of this review was to evaluate renal tumors during pregnancy in terms of epidemiology, risk factors, diagnosis and the safety of a robot-assisted laparoscopic approach in the management of these tumors.

## 2. Study Design and Selection Criteria

**Figure 1:**
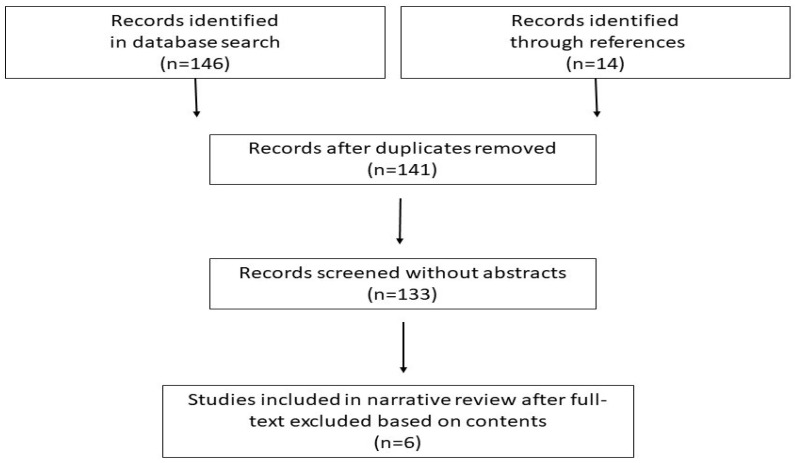
Study flow chart.

On 1 December 2024, a search of medical databases was performed to find articles that included the following keywords: “pregnancy”, “obstetric”, “robotic”, “surgery”, “robot”, “robot-assisted”, “minimally procedures”, “da vinci” or “davinci”, “renal tumor”, “kidney tumor”, “management”, “renal cancer” and “renal cell carcinoma”, “Therapy”. MEDLINE, Web of Science, PubMed and Google Scholar were used to search for eligible articles published in the literature. The search yielded 146 articles. Eligibility of the studies was independently reviewed by two authors (L.D. and V.S.). Studies that addressed non-obstetric transabdominal robotic surgery occurring during pregnancy were included. The main aim of the present narrative review was to identify robotic surgery in the management of renal tumors. Therefore, studies including robotic partial nephrectomy or radical nephrectomy for renal masses were selected for this review. The reference lists of all included studies were examined to help identify studies not captured by the initial search. A total of six case studies were included in the final literature review (Figure 1).

## 3. Epidemiology and Etiology

The diagnosis of cancer during pregnancy is a rare event, with an estimated incidence of 1 in every 1000 pregnancies [9]. Among the most frequently diagnosed gestational cancers are breast cancer, lymphoma, and cervical cancer [10]. Malignant ovarian tumors are less common, comprising approximately 5% of ovarian masses identified during pregnancy [10]. Renal masses detected during pregnancy are also uncommon, with only around 100 cases documented in the literature to date. Of these, approximately 50% are malignant, potentially influenced by hormonal and paracrine effects. Among benign renal masses, angiomyolipomas account for 23%, while oncocytomas are observed in 3–7% of cases [11]. However, the role of pregnancy-related hormonal changes in promoting the formation of malignant kidney cells remains uncertain. Urinary tract cancers during pregnancy are rare, with renal cell carcinoma (RCC) being the most frequently diagnosed urological malignancy. RCC accounts for about 3% of all cancers in the general population, and despite the trend of delayed childbirth, its incidence during pregnancy remains low [3]. The routine use of ultrasonography in antenatal care may contribute to the increased incidental detection of gestational RCC [12]. Fetal metastasis has primarily been reported in cases involving placental metastases, such as melanoma and lung cancer, and is associated with a poor prognosis [13]. Based on an analysis of gestational RCC cases reported since 2004, the median age at diagnosis is 33 years, with patients ranging from 20 to 52 years old [14]. RCC during pregnancy is most commonly diagnosed in the second trimester [12]. Several factors contribute to the development of renal tumors. Non-modifiable risk factors include age, while modifiable factors encompass obesity, smoking, and hypertension [14,15]. Obesity, in particular, has a strong association with RCC. In the VITAL study by Macleod et al. [16], individuals with a body mass index (BMI) of 35 kg/m^2^ or higher exhibited a 71% greater risk of RCC compared to those with a BMI below 25 kg/m^2^. A meta-analysis of 14 studies further revealed a 7% increase in RCC risk for each unit increase in BMI [17]. This connection may stem from obesity-driven processes, such as increased cell proliferation, angiogenesis, and survival, influenced by elevated levels of gonadal steroids, adipokines, and insulin-like growth factors [1]. Hormonal and reproductive factors during pregnancy may also contribute to RCC development [18]. The gestational period is characterized by heightened levels of estrogen and progesterone, both of which have receptors present on normal and malignant renal cells [19]. Parity has been associated with RCC risk in cohort studies, though the exact mechanisms remain unclear. Hypertension is another independent risk factor for renal tumors, affecting about 18% of pregnant individuals diagnosed with the disease [20]. Studies, including the VITAL study, have identified hypertension as significantly increasing RCC risk (HR 1.70; 95% CI, 1.30–2.22) [16]. The combined presence of obesity, hypertension, and type 2 diabetes further amplifies RCC risk, with women exhibiting all three conditions facing a fourfold higher likelihood of developing the disease compared to those without comorbidities [16,21].

## 4. Diagnosis and Imaging

The detection of renal masses during pregnancy often occurs incidentally during ultrasound examinations performed for unrelated reasons, such as investigating routine antenatal ultrasonography or hydronephrosis caused by non-neoplastic factors (e.g., kidney stones or ureteral compression) or ascending urinary tract infections [22]. Common symptoms include pain (50%), hematuria (37%), hypertension (12%), and the classic triad of hematuria, pain, and a palpable mass (21%). Hematuria in pregnancy is typically attributed to non-neoplastic conditions like infections, stones, bladder varices, or glomerulonephritis [23]. Other symptoms, such as fever (21%), weight loss (9%), or hemorrhagic shock due to tumor bleeding (3%), are less frequent, while rare presentations include hemolytic anemia, hypercalcemia, or ruptured tumor cysts. Five cases of metastatic disease during pregnancy have been documented, with most diagnoses occurring in the second or third trimester, while first-trimester cases are less common [24]. Diagnostic imaging during pregnancy is often restricted due to the potential risks associated with ionizing radiation from conventional radiography and CT (Computed Tomography) scans, which can lead to congenital anomalies, growth restrictions, or stillbirth [25,26]. Ultrasound and MRI (Magnetic Resonance Imaging), which are considered safe throughout pregnancy, are the preferred imaging methods. Ultrasound is particularly effective in identifying renal masses larger than 2 cm but has limitations in detecting smaller lesions or those that do not alter kidney contours. For lesions measuring 2 cm or less, CT and ultrasound sensitivity are approximately 94% and 79%, respectively [27]. Both CT and MRI exhibit nearly 100% accuracy for diagnosing renal tumors, with CT being the preferred imaging technique. CT scans that exclude the gravid uterus result in minimal fetal radiation exposure and are generally safe during pregnancy [28]. A single CT scan through the uterus exposes the fetus to a dose of up to 25 mGy, while abdominal or pelvic CTs expose the uterus to 18–25 mGy and are reserved for emergencies [27,28]. Radiation doses exceeding 50–100 mGy within two weeks post-conception may cause fetal loss or have no effect. During organogenesis (weeks 2–8), high doses (>200 mGy) could lead to anomalies, while lower doses (>60 mGy) between weeks 8 and 15 might result in intellectual disabilities or microcephaly [29]. Radiation exposures of 10–20 mGy may double the lifetime risk of leukemia, and fetal thyroid or gonadal damage is a theoretical concern with certain radiation types [30]. MRI provides a reliable, though costlier, alternative to CT for evaluating renal masses in pregnancy, offering detailed information on tumor size, local extension, and potential vascular involvement. While gadolinium, a contrast agent, crosses the placental barrier, no significant fetal harm has been reported, although it could potentially induce fetal hypothyroidism [31]. Whole-body MRI is gaining recognition as a method for cancer staging, with sensitivity comparable to skeletal scintigraphy for detecting bone metastases [32]. However, its ability to detect pulmonary lesions compared to chest CT remains uncertain. The European Society of Radiology supports MRI use throughout pregnancy in all stages [28,31]. The differential diagnosis for renal masses includes inflammatory, cystic, benign, and malignant lesions beyond renal cell carcinoma. Preoperative biopsy to confirm renal mass diagnosis during pregnancy is rare, but tumor core biopsy offers high accuracy in identifying malignancy [33]. A biopsy is considered for indeterminate renal masses, suspected metastasis, or before initiating treatment, although evidence on the safety of ultrasound-guided biopsy during pregnancy is limited. Typically, definitive diagnosis relies on histopathological evaluation of surgical specimens [34,35].

## 5. Robotic Management

**Figure 2:**
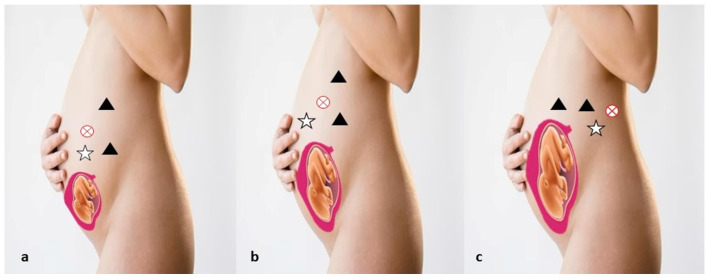
Robotic ports position (black triangle: robotic-8 mm trocar; white star: 12 mm trocar assistant; white circle: robotic-8 mm trocar camera position) in pregnancy during: 1st Trimester (**a**); 2nd Trimester (**b**); 3rd Trimester (**c**).

Urological tumors during pregnancy are uncommon, but early diagnosis can significantly improve outcomes by allowing interventions before tumor progression and when the uterus is smaller [36]. Around 132 cases of renal masses have been documented in the literature, including 93 cases of RCC. Loughlin et al. reviewed the management of solid renal masses in pregnancy, advising radical surgery during the first trimester or after 28 weeks of gestation in the second trimester [37]. For masses diagnosed in the third trimester, surgical timing depends on lung maturity, but for those near term, surgery can be postponed until after delivery. Some experts suggest immediate nephrectomy regardless of pregnancy stage, prioritizing maternal health [14,38,39]. Surgical intervention during the second trimester is generally considered safer, as the risk of spontaneous abortion or congenital abnormalities is higher in the first trimester, while preterm labor is more likely in the third [35,40]. Laparoscopic techniques have transformed minimally invasive surgery, with robotic approaches emerging as a cutting-edge option [3]. Robotic surgery, suitable even during pregnancy, offers advantages like enhanced visualization and precision, particularly when operating alongside a gravid uterus [41]. Our review highlights the limited application of robotic surgery for renal masses in pregnant patients, with only six documented cases [1,42,43,44,45,46] in the last two decades (Table 1). Initial abdominal access can be safely achieved using techniques such as open (Hasson), Veress needle, or optical trocar, adjusted based on uterine size. If the uterine fundus surpasses the umbilicus, upper abdominal access is preferred. In the transperitoneal robot-assisted technique, three robotic ports are aligned para-rectally, with a 12 mm Air Seal port below the umbilicus (Figure 2). CO_2_ insufflation is typically maintained at 8–12 mmHg, although higher pressures may pose risks to uterine blood flow and fetal health [42,43,44,45]. For retroperitoneal approaches, an incision below the 12th rib provides access, with a balloon dissector creating space for port placement. Retroperitoneal techniques involve less uterine irritation, while transperitoneal approaches offer a broader surgical field and better visibility of landmarks [43]. Robotic partial nephrectomy (RPN) is advantageous over laparoscopic methods due to lower conversion rates to radical nephrectomy, better preservation of kidney function, shorter hospital stays, and reduced ischemia times. To date, only five cases of RPN in pregnant patients have been reported. Most occurred during the second trimester with transperitoneal access and controlled CO_2_ pressure. A retroperitoneal approach was used in a third-trimester case to accommodate an enlarged uterus (Figure 2). The right-side supine position must be managed cautiously in late pregnancy to prevent compression of the inferior vena cava [43]. Minimizing CO_2_ insufflation and maintaining intraperitoneal pressure below 12 mmHg can reduce complications. Retroperitoneal approaches further lower risks, offering early control of the renal hilum and reduced bowel manipulation [44].

## 6. Treatment Plan and Outcomes

Managing renal masses during pregnancy is a complex and demanding topic. There is no universal agreement on the optimal timing for interventions or the positioning of the patient during procedures [48]. Any solid renal tumor identified during pregnancy should be presumed to be RCC until proven otherwise [3]. To confirm or exclude this diagnosis, a nephrectomy—either partial or radical—may be required. Rajeev et al. described a case involving a renal mass during pregnancy, where a preoperative fine-needle biopsy suggested an oncocytic renal neoplasm [1]. However, definitive histological analysis later identified it as chromophobe renal cell carcinoma. Following thorough counseling, the patient underwent a robotic radical nephrectomy at 13 weeks of gestation [1,37]. This highlights the importance of recognizing that negative biopsy results do not definitively rule out malignancy. Laparoscopic radical nephrectomy and nephron-sparing surgeries are now commonly utilized to treat renal tumors during pregnancy [1,3]. However, there is an ongoing debate regarding the most appropriate treatment strategy. Surgical interventions during pregnancy require careful monitoring of maternal hemodynamics to ensure adequate utero-placental blood flow and to minimize the risk of preterm labor [49]. Not all renal tumors discovered during pregnancy demand immediate surgical intervention [50,51]. Decisions regarding treatment should consider the health of both the mother and fetus, the aggressiveness of the tumor, and, most importantly, the preferences of the pregnant individual [52,53]. When surgery becomes necessary, the timing is crucial. For localized renal tumors (stage I or II), the general recommendation is to avoid surgical procedures during the first trimester [54,55]. Interventions are typically safer in the second trimester or after fetal lung development is complete. For advanced tumors (stage III or IV), pregnancy termination may be advised to prioritize treatment for the renal tumor. Radical nephrectomy and nephron-sparing surgeries remain critical options for RCC management [56]. The National Comprehensive Cancer Network guidelines suggest that stage I renal masses (T1a) are best managed with partial nephrectomy. For T1b tumors, partial nephrectomy has demonstrated comparable outcomes to radical surgery [57]. Radical nephrectomy is the standard treatment for stages II and III, while partial nephrectomy may be considered in cases of locally advanced tumors if technically feasible [55,57]. Reports indicate that some stage T1 and nearly all stage T2 renal tumors in pregnant patients were treated with radical nephrectomy to reduce operative duration and minimize perioperative risks [38,40]. Studies have shown the feasibility of laparoscopic nephrectomy during pregnancy, with no significant complications [46]. Similarly, robotic surgeries were successfully performed without notable adverse effects. Current evidence suggests no significant increase in the risk of preterm labor or perioperative complications for either mother or child [42,43,44,45].

### Treatment of Metastatic Renal Tumors

Current strategies for managing metastatic renal tumors prioritize combination therapies involving immune checkpoint inhibitors. The standard treatment across all risk groups includes combinations such as pembrolizumab plus axitinib and nivolumab plus ipilimumab. When immune checkpoint inhibitors are unavailable or contraindicated, VEGFR-TKIs can serve as alternative options [2,58]. The safety profile of these treatments during pregnancy is not well-established. Limited research exists, and the available data primarily come from animal studies [59,60]. According to the United States Food and Drug Administration, these medications are classified as Category D, indicating potential risks to the fetus and advising against their use during pregnancy [61]. Consequently, systemic treatment is not advised for pregnant patients with RCC who have undergone radical tumor resection and do not exhibit metastasis. However, for patients with distant metastases, systemic therapy may be considered after pregnancy termination [62].

## 7. Future Directions and Conclusions

Although rare, renal masses identified during pregnancy can be malignant. In cases of renal cell carcinoma during pregnancy, timely diagnosis and appropriate management are vital for optimizing maternal and fetal outcomes. While no specific guidelines for managing renal tumors in pregnant patients currently exist, clinicians can rely on established protocols for kidney tumor treatment in non-pregnant individuals and surgical guidelines tailored to pregnancy. Emerging evidence indicates that routine ultrasonographic screening facilitates the early detection of renal cancer during pregnancy, enabling timely intervention. Radical nephrectomy has demonstrated a high curative potential and can be performed safely during pregnancy with favorable maternal and fetal outcomes comparable to those in non-pregnant patients with renal cancer. Advances in robotic surgery have contributed to reduced blood loss, quicker postoperative recovery, and operation times that align with safety standards for pregnant patients. The advantages of this robotic approach are smaller incisions, leading to lower morbidity, less postoperative pain and shorter hospital stays, which are similar to any minimally invasive surgery. However, robotics do seem to have an edge in highly complicated procedures when extensive dissection and proper anatomy reestablishment are required. The decision regarding the timing and necessity of surgery in pregnant patients requires a careful assessment of maternal health, fetal development, and the progression of the disease. In cases of extensive metastatic disease, termination of the pregnancy may be considered.

A significant recent advancement in surgical procedures focuses on achieving effective outcomes through less invasive techniques. Many conditions can now be treated using minimally invasive or noninvasive methods, often guided by highly advanced imaging technologies [63]. Among these techniques, thermal-based ablations are commonly performed percutaneously under image guidance. Examples include radiofrequency ablation, microwave ablation, cryoablation, and histotripsy [63,64]. These methods rely on either heating or freezing tissue to induce necrosis. However, thermal ablation methods have certain limitations, such as the “heat sink effect” caused by blood flow, which can reduce the size of the treatment area and create challenges in achieving precise margins [65]. Additionally, there is limited scientific evidence supporting their safe use during pregnancy, which remains an area of concern. However, till these newer options are explored, robotic options remain the prime modality for managing renal tumors during pregnancy. Their effective management demands close collaboration between a multidisciplinary team and the patient to ensure individualized care.

## Figures and Tables

**Table 1:** Management in patients affected by RCC during pregnancy and treated with robotic surgery.

Study	Park SY et al. [42]	Ramirez D et al. [43]	Völler M et al. [44]	Tsutsui K et al. [47]	Rajeev S et al. [1]	Dai K et al. [46]
Patients, n.	1	1	1	1	1	1
Age, y	36	35	27	36	23	25
Age of gestation at diagnosis	14th w	20th w	-	5th w	8th w	6th w
Age of gestation at treatment	16th w	21th w	30th w	15th w	13th w	8 and 20th w
Staging Imaging	MRI	MRI	MRI	CT	CT	CT
Kidney Side	Left	Right	Right	Left	Left	Bilateral
Kidney Localization	Middle portion	Upper pole	Lower pole	Upper pole	Lower pole	Lower pole
Renal biopsy	No	No	No	No	Yes	No
Pathologic stage	T1a	T1b	T1b	T1a	T2a	T3a
Treatment	PN	PN	PN	PN	RN	PN bilateral
Surgical approach	T	T	R	T	T	T
Histologic findings	RCC	RCC	CRCC	RCC	CRCC	HLRCC RCC
Adjuvant treatments	No	No	No	No	No	Ipilimumab
Pregnancy outcome	live-birth	live-birth	live-birth	live-birth	live-birth	live-birth

W = week; CT = Computed Tomography; MRI = Magnetic Resonance Imaging; PN = partial nephrectomy; RN = radical nephrectomy; T = transperiteonal; R = retroperitoneal; RCC = renal cell carcinoma; CRCC = chromophobe renal cell carcinoma; HLRCC = Hereditary leiomyomatosis.

## Data Availability

The original contributions presented in the study are included in the article. Further inquiries can be directed to the corresponding author.
